# Can Reverse T3 Assay Be Employed to Guide T4 vs. T4/T3 Therapy in Hypothyroidism?

**DOI:** 10.3389/fendo.2019.00856

**Published:** 2019-12-11

**Authors:** Cristiane Gomes-Lima, Leonard Wartofsky, Kenneth Burman

**Affiliations:** ^1^MedStar Health Research Institute, MedStar Washington Hospital Center, Washington, DC, United States; ^2^Thyroid Cancer Research Unit, MedStar Health Research Institute, Washington Hospital Center, Washington, DC, United States; ^3^Division of Endocrinology, MedStar Washington Hospital Center, Washington, DC, United States

**Keywords:** reverse T3, thyroid hormones, deiodinases, T4/T3 combination, hypothyroidism, combination therapy, deiodinase 3

Among the controversial issues surrounding combination T4/T3 therapy for hypothyroidism is the choice of the best biochemical parameter by which to monitor therapy. This article explores the potential use of reverse T3 (rT3) for this role. Unsubstantiated claims in layman websites have suggested that measurement of rT3 could serve as a useful guide for monitoring combined T4 and T3 replacement therapy. A brief review of aspects of the generation of rT3 from the peripheral metabolism of T4 may place into context the possible utility of rT3 for this role.

## Thyroid Physiology—Deiodinases

**Figure 1:**
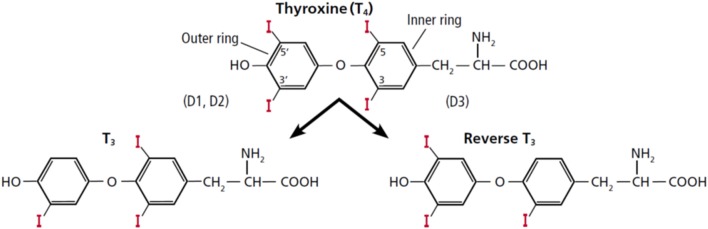
Structure of thyroxine (T4) and the deiodinative cascade to T3 and reverse T3 (rT3). This figure was obtained with permission from Gomes-Lima and Burman (4). Copyright © 2018 The Cleveland Clinic Foundation. All rights reserved.

In humans, a normal thyroid gland produces ~85 mcg of T4 and 6.5 mcg of T3 daily (1). Thus, the ratio of T4:T3 that is directly secreted from the thyroid gland is around 13:1. The remaining daily T3 production, about 26.5 mcg, derives from peripheral monodeiodination or conversion from T4, catalyzed by the activating deiodinases type (D1) or type 2 (D2) (2). D1 catalyzes the deiodination of both the outer and inner ring of T4, while D2 is an obligate outer ring deiodinase (3). Reverse T3 is also generated from deiodination of T4 with production of ~28 mcg/daily, but through deiodination of its inner ring by deiodinase type 3 (D3) (Figure 1). Because rT3 has been considered biologically inactive, deiodinase D3 represents the main physiological inactivator of thyroid hormones. It also mediates the inactivation of T3 into T2. Theoretically, the inactivation of T4 with generation of rT3 or T2 has a homeostatic role by protecting tissues from excess of thyroid hormones. Deiodinases are selenoproteins, accounting for the presence of the rare amino acid selenocysteine (Sec) in their active site (3). Adequate levels of selenium are important to the activity of all deiodinases.

Deiodinases have variable expressions among various tissues (Table 1). In humans, D1 is expressed mainly in the liver, kidneys, thyroid, and pituitary, while D2 is expressed in the central nervous system (CNS), pituitary, brown adipose tissue, thyroid, placenta, skeletal muscle, and heart. D1 is notably absent in the CNS. D3 is present in the skin and in the CNS, and is highly expressed in hemangiomas, fetal liver, placenta and fetal tissues (3). The high expression of D3 in the human placenta and fetal tissues is consistent with the concept that the function of D3 is to limit the exposure of fetal tissues to thyroid hormones.

**Table 1:** Tissue distribution of deiodinases.

**Deiodinase**	**Site of action**	**Resulting action**	**Tissue distribution in humans**
D1	Inner and outer ring of T4	Activating: generates preferably T3	Liver, kidneys, thyroid, and pituitary.
D2	Outer ring of T4	Activating: generates T3	CNS, pituitary, brown adipose tissue, thyroid, placenta, skeletal muscle, and heart.
D3	Inner ring of T4	Inactivating: generates rT3	Hemangiomas, fetal liver, placenta and in fetal tissues; skin and CNS.

Deiodination of the outer ring of T4 by D1 or D2 has been termed the activating pathway of T4 metabolism as it generates the most active thyroid hormone, T3. By this pathway, deiodinases play a critical role in maintaining tissue and cellular thyroid hormone levels of T3, which is then available to bind to hormone binding nuclear receptors and initiate thyroid hormone specific effects. Due to alterations in rates of local deiodination in tissues, thyroid hormone signaling can change irrespective of serum hormonal concentrations (3, 5). Deiodinases also modulate the tissue-specific concentrations of T3 in response to iodine deficiency, hyperthyroidism, and hypothyroidism (3). Thus, during iodine deficiency or hypothyroidism, tissues that express D2, especially the brain, increase the activity of this enzyme to increase local conversion of T4 to T3. In contrast, D1 overexpression in hyperthyroidism contributes to a relative excess of T3 production, while D3 up-regulation in the brain protects the CNS from excessive amounts of thyroid hormone (3).

Although rT3 is not widely expressed in adult tissues, it can be re-expressed in several pathophysiological conditions, such as cancer, starvation, cardiac hypertrophy, myocardial infarction, chronic inflammation, and critical illness (5). In these critical conditions, where a reduction of the metabolism and a reduced T3 level is physiologically desirable, the conversion of T4 to T3 is reduced, while the conversion to rT3 is increased. This is the basis of the alterations in thyroid hormone levels in the euthyroid sick syndrome or non-thyroidal illness syndrome (NTIS) (6). In general terms, this syndrome is characterized by normal or even decreased serum TSH levels in the presence of low serum T3 levels and serum levels of free T4 that may be normal, increased, or decreased. Since thyroid function tests are abnormal but inconsistent with hypothyroidism, it is frequently challenging to decide whether these patients need immediate thyroid replacement therapy. Further confounding the assessment of thyroid function of these patients is the associated frequent administration of drugs such as glucocorticoids, heparin, and dopamine that also influence thyroid hormone economy (6, 7).

**Figure 2:**
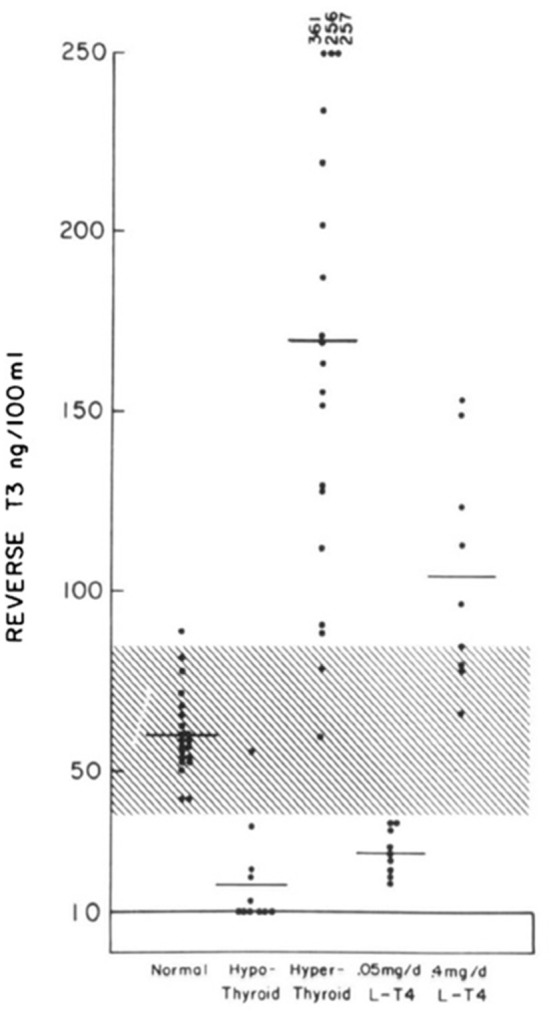
Individual values of serum reverse T3 levels in normal, hypothyroid, and hyperthyroid subjects and in athyreotic patients who have been administered 50 mcg of LT4 and 400 mcg of LT4 daily [This figure was obtained with permission from Burman et al. (9)].

Our understanding of the deiodinative pathways for T4 was accelerated by the development of a specific radioimmunoassay for rT3 (8). Reverse T3 was identified in the serum of normal individuals, in patients with hypothyroidism, hyperthyroidism and athyreotic patients being treated with different doses of levothyroxine (Figure 2) (9). It soon became apparent that the reciprocal changes in serum T3 and rT3 in patients with severe non-thyroidal diseases represented homeostatic attempts to conserve energy (10). However, rT3 has not proved reliable in differentiating euthyroid sick syndrome and hypothyroidism in critically ill patients. One retrospective study demonstrated that variations in rT3 levels may be associated with a variety of thyroid states (11). Therefore, diagnosis of thyroid status and subsequent management of patients with critical illness should be based upon the combined estimation of TSH, free T4 and total T3 serum levels together with clinical parameters and close follow-up of the evolving clinical and laboratory picture (6, 11).

## Clinical Utility of Reverse T3

The early belief that rT3 measurement could be a useful laboratory marker of the euthyroid sick syndrome prompted many physicians to request this assay for patients in the critical care setting. But it soon became clear that in such situations, rT3 levels must be taken into context with other thyroid function tests, namely TSH, free T4, and total T3. Unfortunately, use of the rT3 assay has not clarified the interpretation of thyroid function tests and thyroid status (11).

Of course, the overwhelming majority of patients with a history of thyroid dysfunction are not managed in the critical care setting but in the office. However, even in the outpatient setting, a few clinical situations can present as mild forms of the euthyroid sick syndrome. During fasting or relative caloric deprivation, for instance, serum T3 is decreased as a homeostatic response to conserve energy and protein. A simultaneous increase in rT3 occurs in the first 2 weeks after caloric restriction, followed by normalization. Free T4 levels may be transiently increased or normal, while TSH levels may be normal or decreased (12, 13). Recognition of these laboratory abnormalities is important due to the increasingly popular use of fad diets that may include periods of fasting and deprivation of specific nutrients. Interpretation of the results of rT3 measurements in these patients should be approached with caution.

Before considering the possible use of rT3 to monitor patients on combined T4/T3 therapy vs. T4 treatment alone we should briefly review the apparent justification for such combined drug therapy. Most physicians caring for hypothyroid patients on T4 monotherapy see a significant subset of subjects who still complain of symptoms suggestive of thyroid hormone insufficiency in spite of TSH levels within the reference range. The argument made is that these patients suffer from insufficient T3 generation from T4. To attempt to generate T3 levels equivalent to those seen with thyroidal secretion of T3, the potential role and efficacy of combination T4/T3 treatment has been assessed. Having a blood test like rT3 to successfully address appropriate dosing of a T4/T3 combination agent could allow clinicians to more effectively treat patients with primary hypothyroidism.

The rationale for a need for combined T4/T3 therapy is based in large part on the discovery of polymorphisms in the deiodinase genes resulting in altered set points of feedback regulation of TSH. One common Thr92Ala polymorphism has been associated with insulin resistance, obesity, hypertension and importantly, altered responses to T4 replacement therapy that predicted the need for a higher T4 dosage, although only in thyroidectomized subjects but not in patients with autoimmune hypothyroidism (14–16). Similarly, patients with a rarer CC genotype of the rs225014 polymorphism in the deiodinase 2 gene showed a greater degree of improvement on T4/T3 therapy than on T4 monotherapy (17). Thus, while endocrinologists traditionally rely on TSH levels and TSH “normalization” during L-T4 therapy to reflect euthyroidism in all tissues, these deiodinase polymorphism studies shed some doubt on the validity of this practice for all patients in view of some clinical evidence supporting a role for combination T4/T3 treatment. These observations bring us back to the question as to whether monitoring another parameter of T4 metabolism, i.e., rT3, could better assess thyroid status by allowing us to target the optimal physiologic T4/T3 ratio. The rationale for T4/T3 combination treatment is based on the premise of low activity of D2 in selected patients, which does not necessarily imply higher D3 activity. Therefore, the rationale for the use of rT3 to monitor combination therapy would appear somewhat tenuous. Some studies comparing physiologic effects of combination T3/T4 treatment to L-T4 monotherapy have evaluated other parameters reflective of thyroid status such as serum SHBG or markers of bone turnover (18) but a practical and useful marker has not been identified.

So, can we justify the use of rT3 to guide levothyroxine therapy or levothyroxine (LT4) + liothyronine (LT3) combination therapy in hypothyroidism? There does not appear to be any rationale to measure rT3 to initiate or adjust levothyroxine therapy, and traditionally the best test for these purposes has been TSH measurement. Treatment decisions based on rT3 levels may lead to the use of excessive doses of levothyroxine, resulting in a state of subclinical or even overt hyperthyroidism (4). Moreover, rT3 assays are expensive and not widely available. They may be difficult to interpret depending on the assay used with the more reliable tests employing liquid chromatography/tandem mass spectrometry (LC-MS/MS). For patients who have elected combination therapy, it remains controversial as to what are the best biochemical parameters to monitor therapy. Potential targets include FT4, total T3, FT3, and FT4/FT3 ratio (19). The European Thyroid Association recommends monitoring thyroid function tests before the morning medications, aiming at normal TSH, FT4, FT3, and FT4/FT3 ratio (20). Yet the notable inaccuracy of FT3 assays requires precaution when interpreting serial laboratory results (19, 20). Unfortunately therefore, at the present time there are no data that support for or against the use of rT3 to monitor LT4 + LT3 combination therapy.

It should be mentioned that there is one rare clinical entity for which the measurement of rT3 is essential to determine the correct diagnosis: in consumptive hypothyroidism. This is a rare form of hypothyroidism identified in newborns with infantile hepatic hemangiomatosis (21). It must be considered in the differential diagnosis of congenital hypothyroidism that requires inappropriately higher doses than usual. In 2000, Huang et al. reported the first case of severe hypothyroidism in a child with infantile hemangiomas (22). Despite the use of high doses of prednisolone and levothyroxine, the infant died from systemic complications after embolization of multiple hemangiomas. In that patient, the high serum levels of rT3 (413 ng/mL), followed by the demonstration of high expression of deiodinase type 3 (D3) in hemangioma tissue, were crucial to the diagnosis. This case report opened a new perspective in the understanding of the role of D3 in the euthyroid sick syndrome (23, 24). Understanding the molecular mechanisms that lead to the reactivation of D3 in illness is an important field of research. Several other cases of consumptive hypothyroidism have been reported so far; in adults they are even less common and have been associated with neoplasms (21, 25, 26).

## Conclusion

Reverse T3 is physiologically relevant to thyroid economy. However, its clinical use as a biochemical parameter of thyroid function is very limited. Currently, no evidence supports the use of rT3 to monitor levothyroxine therapy, either given alone or in combination with liothyronine.

## Author Contributions

CG-L wrote the paper with input from LW and KB.

### Conflict of Interest

The authors declare that the research was conducted in the absence of any commercial or financial relationships that could be construed as a potential conflict of interest.

## References

[1] Jonklaas J Bianco AC Bauer AJ Burman KD Cappola AR Celi FS. Guidelines for the treatment of hypothyroidism: prepared by the american thyroid association task force on thyroid hormone replacement. Thyroid. (2014) 24:1670–751. 10.1089/thy.2014.0028 25266247 PMC4267409

[2] Engler D Burger AG. The deiodination of the iodothyronines and of their derivatives in man. Endocr Rev. (1984) 5:151–84. 10.1210/edrv-5-2-151 6376077

[3] Bianco AC Salvatore D Gereben B Berry MJ Larsen PR. Biochemistry, cellular and molecular biology, and physiological roles of the iodothyronine selenodeiodinases. Endocr Rev. (2002) 23:38–89. 10.1210/edrv.23.1.0455 11844744

[4] Gomes-Lima C Burman KD. Reverse T3 or perverse T3? Still puzzling after 40 years. Cleve Clin J Med. (2018) 85:450–5. 10.3949/ccjm.85a.17079 29883303

[5] Dentice M Salvatore D. Deiodinases: the balance of thyroid hormone: local impact of thyroid hormone inactivation. J Endocrinol. (2011) 209:273–82. 10.1530/JOE-11-0002 21398344

[6] Wartofsky L Burman KD. Alterations in thyroid function in patients with systemic illness: the euthyroid sick syndrome. Endocr Rev. (1982) 3:164–217. 10.1210/edrv-3-2-164 6806085

[7] Jaume JC Mendel CM Frost PH Greenspan FS Laughton CW. Extremely low doses of heparin release lipase activity into the plasma and can thereby cause artifactual elevations in the serum-free thyroxine concentration as measured by equilibrium dialysis. Thyroid. (1996) 6:79–83. 10.1089/thy.1996.6.79 8733876

[8] Chopra IJ. A radioimmunoassay for measurement of 3,3',5'-triiodothyronine (reverse T3). J Clin Invest. (1974) 54:583–92. 10.1172/JCI107795 4211761 PMC301591

[9] Burman KD Dimond RC Wright FD Earll JM Bruton J Wartofsky L. A radioimmunoassay for 3,3',5'-L-triiodothyronine (reverse T3): assessment of thyroid gland content and serum measurements in conditions of normal and altered thyroidal economy and following administration of thyrotropin releasing hormone (TRH) and thyrotropin (TSH). J Clin Endocrinol Metab. (1977) 44:660–72. 10.1210/jcem-44-4-660 191466

[10] Burman KD. Recent developments in thyroid hormone metabolism: interpretation and significance of measurements of reverse T3, 3,3'T2, and thyroglobulin. Metabolism. (1978) 27:615–30. 10.1016/0026-0495(78)90028-8 642830

[11] Burmeister LA Reverse T3 does not reliably differentiate hypothyroid sick syndrome from euthyroid sick syndrome. Thyroid. (1995) 5:435–41. 10.1089/thy.1995.5.435 8808092

[12] Spencer CA Lum SM Wilber JF Kaptein EM Nicoloff JT. Dynamics of serum thyrotropin and thyroid hormone changes in fasting. J Clin Endocrinol Metab. (1983) 56:883–8. 10.1210/jcem-56-5-883 6403568

[13] Moura Neto A Zantut-Wittmann DE. Abnormalities of thyroid hormone metabolism during systemic illness: the low T3 syndrome in different clinical settings. Int J Endocrinol. (2016) 2016:2157583. 10.1155/2016/2157583 27803712 PMC5075641

[14] Torlontano M Durante C Torrente I Crocetti U Augello G Ronga G. Type 2 deiodinase polymorphism (threonine 92 alanine) predicts L-thyroxine dose to achieve target thyrotropin levels in thyroidectomized patients. J Clin Endocrinol Metab. (2008) 93:910–3. 10.1210/jc.2007-1067 18073314

[15] Heemstra KA Hoftijzer HC van der Deure WM Peeters RP Fliers E Appelhof BC Thr92Ala polymorphism in the type 2 deiodinase is not associated with T4 dose in athyroid patients or patients with Hashimoto thyroiditis. Clin Endocrinol. (2009) 71:279–83. 10.1111/j.1365-2265.2008.03474.x 19018782

[16] Castagna MG Dentice M Cantara S Ambrosio R Maino F Porcelli T. DIO2 Thr92Ala reduces deiodinase-2 activity and serum-T3 levels in thyroid-deficient patients. J Clin Endocrinol Metab. (2017) 102:1623–30. 10.1210/jc.2016-2587 28324063

[17] Panicker V Saravanan P Vaidya B Evans J Hattersley AT Frayling TM. Common variation in the DIO2 gene predicts baseline psychological well-being and response to combination thyroxine plus triiodothyronine therapy in hypothyroid patients. J Clin Endocrinol Metab. (2009) 94:1623–9. 10.1210/jc.2008-1301 19190113

[18] Schmidt U Nygaard B Jensen EW Kvetny J Jarlov A Faber J. Peripheral markers of thyroid function: the effect of T4 monotherapy vs T4/T3 combination therapy in hypothyroid subjects in a randomized crossover study. Endocr Connect. (2013) 2:55–60. 10.1530/EC-12-0064 23781319 PMC3680960

[19] Jonklaas J. Risks and safety of combination therapy for hypothyroidism. Expert Rev Clin Pharmacol. (2016) 9:1057–67. 10.1080/17512433.2016.1182019 27137849

[20] Wiersinga WM Duntas L Fadeyev V Nygaard B Vanderpump MP. 2012 ETA guidelines: the use of L-T4 + L-T3 in the treatment of hypothyroidism. Eur Thyroid J. (2012) 1:55–71. 10.1159/000339444 24782999 PMC3821467

[21] Luongo C Trivisano L Alfano F Salvatore D. Type 3 deiodinase and consumptive hypothyroidism: a common mechanism for a rare disease. Front Endocrinol. (2013) 4:115. 10.3389/fendo.2013.00115 24027558 PMC3761349

[22] Huang SA Tu HM Harney JW Venihaki M Butte AJ Kozakewich HP. Severe hypothyroidism caused by type 3 iodothyronine deiodinase in infantile hemangiomas. N Engl J Med. (2000) 343:185–9. 10.1056/NEJM200007203430305 10900278

[23] Huang SA Bianco AC. Reawakened interest in type III iodothyronine deiodinase in critical illness and injury. Nat Clin Pract Endocrinol Metab. (2008) 4:148–55. 10.1038/ncpendmet0727 18212764 PMC3133953

[24] Wajner SM Maia AL. New insights toward the acute non-thyroidal illness syndrome. Front Endocrinol. (2012) 3:8. 10.3389/fendo.2012.00008 22654851 PMC3356062

[25] Simsek E Demiral M Gundogdu E. Severe consumptive hypothyroidism caused by multiple infantile hepatic haemangiomas. J Pediatr Endocrinol Metab. (2018) 31:823–7. 10.1515/jpem-2018-0055 29953409

[26] Howard D La Rosa FG Huang S Salvatore D Mulcahey M Sang-Lee J. Consumptive hypothyroidism resulting from hepatic vascular tumors in an athyreotic adult. J Clin Endocrinol Metab. (2011) 96:1966–70. 10.1210/jc.2010-2104 21508133 PMC3135192

